# Walking and Sitting Outdoors: Which Is Better for Cognitive Performance and Mental States?

**DOI:** 10.3390/ijerph192416638

**Published:** 2022-12-11

**Authors:** Andrew W. Bailey, Hyoung-Kil Kang

**Affiliations:** 1 Department of Health and Human Performance, The University of Tennessee at Chattanooga, Chattanooga, TN 37403, USA; 2 Department of Physical Education, Kyungnam University, Changwon 51767, Republic of Korea

**Keywords:** natural environment, physical activity, EEG, cognitive performance, environmental psychology, mental states

## Abstract

Myriad research indicates that physical activity and natural environments enhance cognitive performance and mental health. Much of this research is cross-sectional or involves physical activity in outdoor environments, rendering it difficult to ascribe the results to a particular condition. This study utilized electroencephalography (EEG) and established cognitive performance tasks to determine the impact of a short intervention including either walking or sitting in an outdoor environment. In this experiment, a total of 50 participants were randomized into walking and sitting groups, with cognitive performance measured before, after, and 10 min post intervention. Both groups demonstrated improvements in cognitive performance, with no significant difference between groups. Elevated levels of relaxation during the intervention were the best predictor of post-test performance. Participants reporting a higher connection to nature, as well as state-based mindfulness during the outdoor intervention, also reported lower levels of frontal theta (i.e., rumination) during the interaction, while the walking group demonstrated higher relaxation. These findings provide a direct connection to neural mechanisms influenced by physical activity and the natural environment, and their impact on cognitive performance. This supports Attention Restoration Theory and the effectiveness of short outdoor interventions incorporating physical activity as a method of restoring mental attention.

## 1. Introduction

Acute and routine participation in physical activity (PA) demonstrates consistent connections to cognitive performance and can be a robust preventer of physical and mental illness. Regular PA boosts the immune system, reduces the risk of cardiovascular disease and diabetes, and lowers the risk of common forms of cancer [1,2]. Physical activity can improve mood, reduce depressive symptoms, and prevent the onset of dementia in older adults [3]. Though current Centers for Disease Control & Prevention (CDC) guidelines recommend five hours of moderate to vigorous PA per week, physical and mental benefits can be reaped at much lower levels. Johnson et al. [4], for instance, found that low-intensity PA was a strong predictor of cognitive performance in older adults. Gothe [5] confirmed those results for African American adults, while adding that light and moderate PA also improve reaction times on cognitive tests. It appears that any PA is better than none, even if deficient of recommended guidelines.

Empirical associations of PA and positive health metrics abound, though the mechanisms of influence remain obscure. Research on animals and humans has demonstrated that PA induces both immediate and long-term changes in structure and function of the brain. Higher levels of PA are associated with increased gray matter in the frontal and hippocampal regions of the cortex in children, which are associated with problem-solving and memory [6]. Even short durations of PA can increase blood flow to all cortical regions, carrying oxygen and nutrients to enhance optimal function [7]. Researchers have identified improvements in neural network efficiency and structural changes through epigenetics due to PA [8,9]. These bio-physiological shifts translate into improved cognitive abilities, higher test scores [10], and psychological benefits such as decreased depression and anxiety and increased self-efficacy, locus of control, and emotional stability [2,11,12].

Longer-term, structural neuronal changes require chronic PA, though immediate physiological changes from acute exercise can produce similar short-term results [2]. Increases in mood, relaxation and attentiveness have been noted after short (10-min) walks or bike rides, with associated improvements in cognitive performance and reaction time [13,14]. The positive impacts of acute and chronic PA on mental well-being are well-documented, though often conflated with influences from the natural environment.

### 1.1. Natural Environment

Many of the health benefits attributed to PA have also been associated with time spent in natural environments. Enhanced immune systems, enhanced mood, fewer symptoms of depression and anxiety, and improved cognitive performance represent common outcomes ascribed to acute or chronic visits to natural environments, with varying levels of immersion and duration [15]. Though certain natural elements (i.e., water features, tree canopy) produce stronger impacts, the positive influences have been observed in myriad environments, including parks and gardens, indoor environments with plants and windows, as well as virtual reality [16,17,18]. No universal recommendations exist regarding minimal outdoor time needed for positive mental health, though researchers have posited that five hours a month should be the requisite minimum [19]. Environmental research often investigates participants while viewing park-like environments in a sedentary position, not incorporating PA into the model. Though outcome-based studies abound, an understanding of the process inducing change remains elusive. The mechanisms behind the environmental influence are perhaps more dubious than for PA, though theorists have proposed evolutionary, bio-physiological, and psychological rationales.

The Biophilia hypothesis [20] asserts that humans evolved over millennia in a close relationship with the natural world. With brains and bodies wired for optimal performance in a dynamic setting, our modern sedentary and hyper-focused lifestyles induce ailments resulting from the lack of appropriate stimuli and routines. A preference for natural environments full of living things represents an archetype embedded in our DNA. Though difficult to prove, this hypothesis is not at odds with bio-physiological models, which demonstrate human responses to various environments. Prolific research on forest bathing, for example, has shown reduced sympathetic and increased parasympathetic nervous system responses as a result of time spent in natural environments, including reductions in heart rate, blood pressure, cortisol and adrenaline levels, physical pain, and changes in brain activity conducive to relaxation [21]. Encephalographic (EEG) research has confirmed an immediate parasympathetic response when encountering parks on an urban walk, evidenced by higher amplitude alpha and theta wave oscillations and decreased “busy brain” signals such as beta and gamma [22,23]. Indirect interactions, such as nature photographs, videos, and virtual scenery also engender positive results [15], though full nature immersion may amplify impacts through temperature changes, auditory stimulus, and plant-based aerosols [16]. The perception of such changes, however, may require a situational awareness akin to state-based mindfulness.

Physiological and neurological responses to natural environments may come through psychological shifts activated by unique stimuli in a novel setting. As proffered by advocates of Attention Restoration Theory [15,24], nature represents a milieu that contrasts with busy urban life, compelling relaxation and reflection via “soft fascination.” Shaping clouds and fluttering leaves attract one’s attention without dominating thoughts. This facilitates mental relaxation, triggering a parasympathetic response that restores mental energy and cognitive capacities. Such positive responses, though common, are not guaranteed. U.S. citizens spend an average of 93% of their lives indoors [25], resulting in routine alienation from the natural world. As such, the dynamic, unpredictable character of the natural world could induce stress rather than relaxation.

### 1.2. Cognitive Performance and Mental Health

Cognitive function is dependent on an individual’s ability to focus on a task, prioritize relevant information, and persist to successful completion [12,26]. The exercise of executive control using top-down mental functioning is evidenced by activation of the frontal and parietal lobes of the cortex; the frontoparietal network [27]. Use of this system promotes heightened focus to direct attention toward relevant information [27]. Over-activation of executive processes, through daily demands of mental work or constant engagement with media [28], can lead to symptoms of a “busy brain”, resulting in long-term mental fatigue, anxiety, and even depression [29]. While isolation and under-stimulation can also engender cognitive decline, concerns due to rapidly increasing urbanization and associated anxiety disorders render this topic relevant and urgent [30]. The relationship between cognitive performance and mental health is well-established, and has been shown to intensify with age [31]. The lack of natural interactions, especially among youth, has sounded alarms from many researchers [32]. PA and restorative environments promote mental relaxation, providing a regenerative effect that may enhance attentional control post-experience [33]. Given the collinearity of the impacts for PA and natural settings, our study employed a randomized, experimental process to elucidate unique effects. The purpose of this study was to determine the cognitive and neurological impacts of resting versus light physical activity in the same natural environment. A secondary purpose was to determine the influence of connectedness to nature and state-based mindfulness on one’s experience outdoors.

## 2. Materials and Methods

### 2.1. Participants and Procedures

The study sample consisted of 50 students at a mid-sized university in the southeastern United States (The University of Tennessee, Chattanooga). Fifteen were female (28%), 21 (42%) were Black or Latino, and the Mean age was 21. Ten students declined to participate or were unavailable at the time of data collection. The students volunteered to aid with this research as part of a course in sports and recreation management. The participants had no prior knowledge of the study principles or premises, though the results were presented as a learning opportunity after the study completion. Students completed surveys before cognitive and EEG data collection, to account for differences in daily routines and connectedness to nature.

On the date of field-based data collection, students reported in small groups to an interior room in the campus library to complete the hour-long process in turns. Given 14 complete equipment setups (EEG devices, fitness trackers, cell phones), the research was administered within four hours on the same day, to ensure consistency of weather conditions. After being fitted with EEG headsets and fitness trackers, the students were seated and provided with cell phones, which collected neuro-physio data, as well as providing a personal screen from which to complete the cognitive test (Encephelapp^®^). As required by the application developer [34], students completed five complete test runs, to ensure that novelty of the program and the test did not interfere with reliability. They then completed a scored test and were guided outside to a park-like quad on campus.

The natural environment for this study included a large, grassy area located at the center of campus. The rectangular field is immediately surrounded by small ornamental trees, though larger oaks and evergreens loom further away. There is a concrete walking area around the field, measuring ¼ mile long. The study was completed on a comfortable autumn day, with temperatures around 74 degrees F. As with any park-like setting, others were enjoying the space for recreation, relaxation, and reading. Participants were previously randomized into a walking group and a resting group, using the attendance list for each of the four sessions. Walkers spent 10 min circling the green space at a comfortable pace, while sitters were distributed around the area in dispersed seating. This level of physical activity would be considered moderate, as indicated by the CDC guidelines of “walking for pleasure,” or “walking to class” [35]. This intensity level was chosen for its direct applicability to real-world settings (i.e., walking for pleasure in a park) and to support CDC guidelines for a highly accessible activity. Participants were instructed not to talk, but to enjoy the scenery and a brief moment of downtime.

Upon completion of the experimental portion of the study, students then returned to the library location and completed another scored Stroop test. Next, to determine longevity of restorative impact, all students watched a 10 min recorded lecture. The lecture represented a real-world mental challenge that has been shown to deplete mental attention. Finally, the students completed a third Stroop test and the assessment was concluded. After completion of the final Stroop test and removal of equipment, students completed a survey to measure their self-reported experience of state-mindfulness during the outdoor portion of the study. This was included to determine their personal reflection on the experience of the specific outdoor environment under investigation.

### 2.2. Measure

Cognitive performance was assessed using the Stroop task, distributed on cell phones via the EncephelApp^®^ [36]. The Stroop task has been used for decades to measure mental speed and acuity based on responses to congruent and incongruent stimuli [36]. Participants are required to select the color written on the screen and hit the appropriate button as quickly as possible. The first portion of the test presents congruent information, with the word “Green” spelled out in green letters. The Stroop effect is employed in the second, incongruent phase, when the word “Green” could appear in any color, the appropriate response still being “Green”. This incongruent information causes a delay that effects attention, information processing, and reaction time. Participants are scored on accuracy and response time.

The EncephalApp^®^ cell phone application has been verified as a reliable method of measuring cognitive speed and flexibility in various populations [37]. The app demonstrates acceptable test/retest reliability (95% CI: 0.65–0.92, *p* < 0.001)) with resistance to improvements due to instrument bias (i.e., learning how to take the test). Instructions for use are first read aloud to participants, then they are guided through five full practice rounds to ensure they understand the purpose and functions of the test. Once they have been oriented to the test, they then complete five full rounds of the test with the Stroop-off, followed by five more with the Stroop effect activated. Scores are recorded on the cell phone with a time stamp which can be associated with EEG data for analysis.

EEG data were collected using Emotiv Insight portable headsets and a custom iPhone application. Emotiv headsets have been verified for use as research-quality devices, and many studies have utilized their functionality for research [37]. The Insight headset incorporates five passive sensors, measuring brain activity at a rate of 128 Hz on both hemispheres of the frontal lobe (af3 & af4) and temporal lobes (t7 & t8), with a single sensor on the center of the parietal lobe (pz). These locations adhere to the universal 10–20 system, providing information from major cortical regions involved in higher-order processing. The data are pre-filtered with low and high-pass filters to exclude raw data that did not fall within the range of 3–43 Hz, as well as a 5th order sinc filter, and notch filters at 50 Hz and 60 Hz. Data were then converted into discrete oscillatory bandwidths through Fast Fourier Transformation (FFT). Four bandwidths were utilized in this study, including theta (θ), alpha (α), beta (β), and gamma (γ).

Oscillatory frequencies were then transformed into mental states, using formulas established by previous research. External attention was measured using the beta/alpha ratio across the frontal lobe (af3 + af4). This mental state indicates focused attention on an external stimulus (i.e., a lecture) which can deplete mental energy over time. Such focus is appropriate in circumstances requiring higher-order processing, but high beta amplitudes during rest can indicate stress or rumination [29]. Motivation was measured using frontal asymmetry (M = α/β af4 - α/β af3), which has been widely used as a proxy for valence and interest. High frequency oscillations in the parietal lobe were indicative of sensory arousal (A = γ pz), a measure also used for state anxiety [38] depending on the context of the setting. Inward Attention was indicated by high amplitude theta in the frontal lobes and alpha in the parietal lobe (IA = [θaf3 + θaf3]/αpz). Inward attention activates inhibitory filtering of stimuli to maintain cognitive control [27] and can aid with mindfulness meditation [39]. Finally, relaxation was assessed as global alpha amplitudes across all sensors, with alpha indicating an idling mind prepared for action [29]. EEG indicators were tracked throughout the entire process.

Other variables were tracked via survey instruments, to determine their influence on mental states and cognitive performance. Mindfulness was assessed using Tanay and Berstein’s [39] State Mindfulness Scale (SMS). This 21-item scale measures the level of experienced mindfulness over a bounded time period, with subscales for bodily (i.e., “I noticed various sensations caused by my surroundings”) and mental mindfulness (i.e., “I felt closely connected to the present moment”). The SMS was included to determine the level of experienced mindfulness in the environment and conditions provided. The Connectedness to Nature Scale [40] was utilized to assess the personal relationship of each student to the natural environment with 14-items (i.e., “I think of the natural world as a community to which I belong”). This measure was included to account for emotional and communal ties to natural spaces that may influence neurological reactions and was completed prior to other data collection.

### 2.3. Analyses

Changes in Stroop task scores were assessed using repeated-measures analysis of variance (RM ANOVA) with the sit/walk condition as a fixed grouping variable, and simple post hoc analysis to determine differences from timepoint one, to timepoints two and three. EEG data were divided into time-bound epochs prior to each Stroop task, including: (1) Baseline, (2) Sit/Walk condition and (3) Lecture. Each mental state was analyzed separately, using repeated measures analysis of variance (RM ANOVA) with condition as a grouping variable. Repeated post hoc analysis was also included to determine differences between each timepoint. Finally, Pearson correlations and multiple analysis of variance (MANOVA) were utilized to elucidate the influence of survey instruments on mental states during the sit/walk condition. All data were analyzed in SPSS, with ANOVA being utilized for parsimonious comparison of epochs. While this overlooks dynamic changes within each time-bound epoch, it renders immediately interpretable results for comparison with similar research.

## 3. Results

RM ANOVA revealed significant improvements in cognitive performance for both groups after outdoor time, with no additional influence observed for the sitting or walking condition. Post hoc analyses indicated that post-condition Stroop scores improved significantly for both groups and remained unchanged at the follow-up measure after a 10 min lecture. These results can be seen in Table 1, where Mean scores represent time to complete the task (with lower scores indicating more efficiency and fewer errors). A 10 min outdoor break improved immediate cognitive performance by 5%, or an effect size of 0.364 (Cohen’s d). This improvement was maintained for at least ten minutes or 100% of the intervention time.

Significant changes in mental states were observed in all participants over the course of the outdoor condition, though trajectories differed by group. As illustrated in Table 2, frontal beta remained largely unchanged for the sitting group over the course of the study. The walking group, however, demonstrated a significant drop in external attention during the condition, followed by a strong rebound above baseline levels during the lecture. Arousal followed a nearly identical trajectory for both groups, albeit showing a smaller rebound for the walking group during the lecture. This suggests a unique level of frontoparietal deactivation induced by the walking condition. Trajectories for Relaxation (i.e., global alpha) confirm this influence of physical activity. Both groups presented equal alpha amplitudes at baseline and during the lecture, though walkers demonstrated a 1600% increase in global alpha while sitters presented a fraction of that change (90%). Inward attention increased for both groups during the condition, though only reaching marginal significance (*p* = 0.086). There were no significant changes in motivation across the three timepoints.

To determine if changes in mental state during the outdoor condition impacted subsequent cognitive scores, a post hoc ANOVA was conducted with all mental states predicting post-condition Stroop Task scores. Relaxation during the condition was the only EEG measure to impact cognitive function after time in the outdoor setting (F = 5.830, *p* = 0.021, η_p_^2^ = 0.15). A post hoc investigation also revealed that no baseline EEG scores, including relaxation, impacted post-condition Stroop scores. Thus, relaxation during the outdoor condition (i.e., state-based EEG), and not trait-based EEG measures, was a predictor of cognitive performance. Fitness trackers confirmed a small, related change in physiological conditions, with walkers averaging a 13% increase in heart rate, while sitters had a 5% decrease during the condition.

Connectedness to nature and the experience of mindfulness during the outdoor condition demonstrated a robust bivariate correlation (r = 610, *p* < 0.001), suggesting that a positive relationship to nature may facilitate positive experiences in outdoor environments. A final MANOVA was conducted to determine the influence of the CNS (F = 4.812, *p* = 0.036, η_p_^2^ = 0.010) and SMS (F = 8.318, *p* = 0.007, η_p_^2^ = 0.206) on mental states during the outdoor condition, with both scales predicting lower frontal beta amplitudes during the outdoor experience. This finding is consistent with mental downshifting through soft fascination and lends further support for frontoparietal deactivation and relaxation as a key component of mental restoration.

## 4. Discussion

The results from this study confirm the influence of natural encounters on cognitive performance while elucidating the mechanisms of that influence. Both sedentary and walking participants alike demonstrated improvements in cognitive performance after a 10 min outdoor intervention. The effect size was similar to that reported in previous research [41,42], though light physical activity did not magnify the impact. Physical activity has been shown to improve cognitive function and/or delay degeneration, across various levels of frequency and intensity [5,13,43,44]. Much previous research was cross-sectional or included interventions that incorporate physical activity in outdoor environments, rendering it difficult to parcel out the effects. Additionally, the focus of much previous research was the prevention of cognitive decline in aging and sedentary populations [5,45]. The sample in this study was a younger, more active group of college students whose routine physical activity is likely higher than the general population. It is possible that routine activity confounds the impacts of short, acute physical interventions. It is also feasible that the outdoor environment and physical activity have equivalent short-term impacts on cognitive performance. Future research will need to be done incorporating various environments and various levels of physical activity to provide a complete understanding of this phenomenon.

The 10 min outdoor “dosage” provided equivalent effect sizes as those reported in previous outdoor research [46] and by pharmaceutical research for ADHD medications [47]. While our study was not a double-blind experiment, the findings are consistent with a growing body of research supporting the power of natural environments to quiet the mind and improve cognitive performance [13,15]. This study provides further confirmation of the duration of a 10 min dose of nature, by demonstrating consistency of performance after a mentally draining 10 min lecture. Lectures, proofreading, studying and like activities require directed attention that induces mental fatigue [24,48]. While debate exists regarding human attention spans [49], mental fatigue is viewed as a protective response that may be overridden at our own peril [48]. Brief mental respites in conducive environments (and potentially incorporating low-to-moderate intensity physical activity) can restore attentional capacities to enhance immediate performance, maintaining those gains for at least the equivalent amount of time. Future research can improve our understanding of dosage and duration with multiple post-intervention tests to determine longevity of impact. Acknowledgement of best practices for mental restoration, as well as incentivizing and removing barriers to such activities, can improve cognitive performance, translating to better academic achievement, worker productivity, and mental health. Commonly, mental breaks consist of shifting one’s attention to social media, while remaining sedentary in an indoor environment, all of which further deplete mental attention and interfere with cognitive function [23].

Changes in mental state over the course of the experience confirm previous research and inform our understanding of the mechanisms behind cognitive performance. Consistent with previous EEG research, natural environments induced higher relaxation and inward attention [22,42]. Unlike the cognitive performance results, however, physical activity did magnify the effect for EEG indices. Walkers demonstrated clear indicators of frontoparietal deactivation, evidenced by strong increases in global alpha, commensurate with significant decreases in frontal beta (i.e., external attention) and sensory arousal. Increased alpha both during and after exercise has been reported by myriad studies [2,45], and a boost of frontal theta (i.e., inward attention) is supportive of walking as a facilitator of creativity [50]. Alpha waves are indicative of a rested, but alert mind, and post-condition cognitive performance was directly impacted by relaxation during the condition. The empirical connection of elevated alpha to better cognitive performance lends credibility to the Attention Restoration Theory [24]. It is, of course, conceptually rational to conclude that mental deactivation would enhance attentional capacities. Empirical support for the mechanisms impacting mental restoration, though, have been elusive. Post hoc analyses revealed that lower frontal beta, combined with higher alpha and theta at baseline also predicted better cognitive performance on the pre-test (*F* = 3.451, *p* = 0.043, *R*^2^ = 0.16), but baseline EEG did not impact post-test cognitive performance. No individual EEG states at baseline impacted pre-test scores, but relaxation during the outdoor time did lead to post-test performance. EEG indicators are highly correlated over time, demonstrating a trait-like stability. Evidence demonstrating malleability in EEG measures, and associated performance enhancement, is promising for intervention research.

The lack of equivalent EEG changes for the non-walkers requires further consideration. Both groups improved in post-test cognitive performance and both groups demonstrated increases in relaxation and inward attention during the condition, though walkers showed a stronger impact. Non-walkers demonstrated no change in external attention and arousal, indicators of frontoparietal activation. Multiple factors could have contributed to this discrepancy. First, the environment utilized in this study was park-like, but not novel. “Being away” from one’s normal routine is a key component of Attention Restoration Theory, and the use of a campus-based outdoor area may have precluded significant impacts. Other variables in the study also help clarify the effects. Connectedness to nature and state-based mindfulness both reduced frontal beta amplitudes during the outdoor condition, regardless of whether participants were walking or sitting. Elevated frontal beta while at rest is an indicator of rumination and a busy brain [29]. Feeling related to the natural world may enhance one’s ability to be more present in the moment and reap the benefits of outdoor environments. Thus, a portion of the variance in both EEG and cognitive performance can be attributed to one’s ability to disconnect from routine stressors and allow the natural world to induce mental restoration.

The results of this study, while instructive, should be interpreted within the scope of its limitations. While the sample size was adequate for EEG research, ANOVA and other analyses would benefit from greater power afforded by more participants. The sample was randomized into groups, but a repeated-measures study where all participants did both conditions would be more rigorous. The outdoor environment in this study was not novel, nor could it be considered “wild.” Given that less manicured spaces render more potent restorative impacts, a more remote location may have been better suited for the condition. A variety of conditions (i.e., indoor sitting, moderate and vigorous physical activity, longer-term follow-up tests, etc.) would provide a better understanding of dosage and durability of the effect. Finally, though 10 min a day would meet recommended monthly requirements for outdoor time, longer interventions would likely compound research outcomes. Understanding these limitations, the results from this study add unique findings to the body of literature investigating the impacts of physical activity in natural environments.

## 5. Conclusions

Both resting and light to moderate physical activity in natural environments can influence cognitive test scores after a 10 min intervention. Though no additional cognitive influence was found for physical activity in this study, those who were active demonstrated higher levels of mental deactivation, which was associated with better post-test scores. The influence of the intervention lasted at least 10 min beyond the post-test. Finally, those participants who reported stronger connections to the natural environment also experienced more mindfulness during the outdoor intervention, and had lower frontal beta (i.e., rumination) during the experience. These findings support previous research on the positive impacts of outdoor environments and physical activity on mental health indicators and provide evidence for the efficacy of routine short interventions for cognitive performance. Resting outdoors can enhance mental performance, while light to moderate physical activity can greatly enhance mental deactivation for added restoration. Programmers and health providers should include such accessible, cost-effective interventions as part of their arsenal to combat mental distress and decline.

## Figures and Tables

**Table 1:** RM ANOVA results for cognitive test scores over three time points.

RM ANOVA	Timepoints	Begin Mean	End Mean	*F*	*p*	*Partial Eta^2^*
Cognitive Performance (All Participants)	Time 1 vs. Time 2	66.99	63.74	22.614	<0.001 *	0.320
	Time 2 vs. Time 3	63.74	63.07	0.992	0.324	0.020
Cognitive Perfo. × Group	Sit T1 vs. T2	70.24	66.92	0.009	0.924	0.000
	Walk T1 vs. T2	63.98	60.79			
	Sit T2 vs. T3	66.92	65.67	0.669	0.418	0.014
	Walk T2 vs. T3	60.79	60.67			

* Significantly different from baseline.

**Table 2:** RM ANOVA for changes in EEG over three epochs.

	Sit Group		Walk Group		Total		Time × Group	
	*Mean*	*SD*	*Mean*	*SD*	*Mean*	*SD*	*F*	*Partial Eta^2^*
Baseline External Att.	1.28	0.40	1.32	0.71	1.30	0.58	-	-
Baseline Motivation	0.17	0.55	−0.02	0.40	0.07	0.48	-	-
Baseline Arousal	0.79	0.91	1.53	2.93	1.17	2.20	-	-
Baseline Inward Att.	38.76	83.68	14.42	12.51	26.27	59.56	-	-
Baseline Relaxation	1984.30	4528.16	1502.34	3261.88	1737.14	3884.84	-	-
Condition External Att.	1.38	0.64	0.90	0.29	1.13	0.54	6.138 *	0.157
Condition Motivation	0.05	0.16	−0.08	0.39	−0.02	0.30	0.365	0.011
Condition Arousal	1.09	1.45	0.09	0.07	0.58	1.12	5.796 *	0.149
Condition Inward Att.	56.45	154.93	46.68	35.12	51.44	109.59	3.075	0.085
Condition Relaxation	3784.08	10,251.70	35,294.04	43,279.61	19,943.03 *	35,226.82	10.432 *	0.240
Lecture External Att.	1.26	0.47	1.61	0.69	1.45	0.61	1.027	0.038
Lecture Motivation	−0.10	0.42	−0.25	0.82	−0.18	0.66	0.323	0.010
Lecture Arousal	0.49	0.27	0.79	0.71	0.65	0.56	0.385	0.012
Lecture Inward Att.	19.98	14.86	16.32	25.84	18.05	21.16	1.122	0.033
Lecture Relaxation	3377.61	5871.01	3661.70	12641.43	3527.54	9897.66	0.161	0.005

* Significantly Different from Baseline.

## Data Availability

Not applicable.
